# Use of 3D foot and ankle puzzle enhances student understanding of the skeletal anatomy in the early years of medical school

**DOI:** 10.1007/s00276-024-03439-1

**Published:** 2024-07-26

**Authors:** Sami A. Al-Ani, Danny Chandla, John Delieu, Sinling Tiffany Yu, Antonio Fratini, Renia Gkountiou, Claire J. Stocker

**Affiliations:** 1 Aston Medical School, Birmingham, B4 7ET UK; 2 College of Engineering and Physical Sciences, Birmingham, B4 7ET UK

**Keywords:** 3D printing, 3D puzzle, Gamification, Foot and ankle, Teaching anatomy, Active learning

## Abstract

**Purpose:**

3D visualization is an important part of learning anatomy with cadavers generally used to effectuate this. However, high cost, ethical considerations, and limited accessibility can often limit the suitability of cadavers as teaching tools. Anatomical 3D printed models offer an alternative tool for teaching gross anatomy due to their low cost and accessibility. This study aims to investigate if combing gamification with 3D printed models can enhance the learning experience and be effective for teaching anatomy.

**Methods:**

3D printed models of the bones of the foot and ankle were generated, and 267 first-year medical students from 2 consecutive cohorts worked in groups to put it together as a puzzle. Participants completed a questionnaire regarding perceptions of 3D models and their knowledge of foot anatomy, before and after the session and were asked to provide comments.

**Results:**

Analysis of the responses showed a significant increase in the confidence of the learners in their anatomy knowledge and an increased appreciation of the role that 3D models have in enhancing the learning experience. After the session, there were many comments saying how enjoyable and engaging 3D models were.

**Conclusion:**

Through the puzzle element of the session, the students were challenged mentally to work out the anatomical features of the foot and ankle. The combined elements of the puzzle and the features of the 3D model assembly made the activity fun and conducive to active learning. The possibility of having fun was not something the students had considered before the session.

**Supplementary Information:**

The online version contains supplementary material available at 10.1007/s00276-024-03439-1.

## Introduction

Anatomical knowledge underpins the practice of healthcare professions and 3D visuospatial appreciation is an important part of medical and veterinary education [21]. However, while cadaveric specimens are used to demonstrate realistic anatomy, high cost, reforms in medical education, ethical considerations, and limited accessibility can limit their availability and suitability for use in teaching [10, 28]. Web deployable anatomical simulations or "virtual reality learning objects" have been adopted as a substitute, but their use for online and mobile learning is being limited by the declining support for web browser plug-ins for personal computers and unavailability on popular mobile devices like Apple iPad and Android tablets. Widespread application of Virtual/ Augmented reality is relatively restricted, largely due to the high cost and limited accessibility associated with the necessary software and hardware, as well as the interdisciplinarity required to develop these tools [10, 37, 41]. And, while HTML5 VR learning objects would make VR learning more accessible across devices, these are in the early stages of development [30]. Moreover, questions have been raised about the reproducibility of augmented reality learning tools on a larger scale [10].

Anatomical physical models offer an effective tool for understanding the spatial arrangements of anatomical organs and teaching gross anatomy due to their easy accessibility and educational effectiveness. Such models could be a practical tool to bring up the learners' level of gross anatomy knowledge at a low cost [39]. Three-dimensional (3D) printing technology has advanced greatly over the past decade and has many applications in the field of medicine. Orthopaedic surgeons have demonstrated that 3D printing technology can improve patient care and physician education and that this technology can be used to improve surgical techniques, plan for difficult surgeries, and create patient-specific instrumentation and implants [13]. In addition, 3D printing is being used in tissue/organ fabrication, creation of customised prosthetics and implants, medical teaching/training, including procedural skill acquisition and patient-physician communication [24]. A recent review of the role of 3D printed anatomical models in teaching human anatomy, suggested that in the test of anatomical knowledge, the post-training test results from 3D models were higher than those in the cadaver or 2D training and that more students were satisfied with their learning [40].

Cases such as foot and ankle pathology can be complex, with the 3D anatomy challenging to appreciate [13]. Deformity can occur in several planes simultaneously and appreciating complex 3D spatial relationships requires a strong foundational understanding of anatomy and mental 3D visualization skills [21]. Physical models that can be manipulated in a 3D space can significantly benefit the visuospatial understanding of structures with complex spatial relationships [21]. Models can accurately demonstrate bony foot and ankle anatomy [10]. Some have proposed that colour and multi-material 3D printed anatomical models can have the same visual and tactile properties as anatomical specimens and could therefore complement or supplement them in anatomy teaching to compensate for the shortage of cadavers [28]. Moreover, whilst there is a need for studies to investigate the effectiveness of 3D printing in comparison to cadavers, there is also need for research to explore if 3D printing is effective as a supplementary tool in a blended anatomy learning approach [6].

Avoiding models and using computers is a possibility. However, a study found that although computer-based learning resources, including virtual reality, can have a positive impact on learning outcomes, they appear to have significant limitations in comparison to physical models, particularly in areas where the anatomy is complex and students have a lower spatial ability [14]. The authors warn against assuming that greater control and interactivity of computer-based modalities would lead to better learning. We believe combining a physical model with gaming avoids these problems and benefits from the advantages of both of these learning aids.

3D model printing has its own difficulties. Existing segmentation software tools are variable, and segmentation requires experienced readers, is time-consuming and prone to intra-and inter-observer variability [31, 34]. Although obtaining accurate and consistent 3D shape segmentation is challenging, even for human workers, novel semi-supervised learning approaches have been proposed and show promising results accuracy and consistency [27]. Research is required to determine the extent to which alternatives to cadaveric anatomy accurately demonstrate the anatomy and ascertain whether they are effective for teaching anatomy [10].

Several reviews, including meta-analyses, have consistently found that the use of games for educational purposes, promote learning and/or reduce instructional time across multiple disciplines and ages [32]. Several theories have been proposed to explain why games are effective learning tools, but essentially play is a primary socialization and learning mechanism common to all human cultures and many animal species [32]. A study found that an interactive game, which allowed users to manipulate a virtual anatomical 3D model of part of the nervous system, provided a greater understanding of the complex structures [35].

There is lack of uniformity in the definition of gamification [33]. The terms gamification, game-based learning and serious games are sometimes used interchangeably, for some authors they mean slightly different things: Gamification is the application of game concepts (e.g. points, leader boards, prizes) to the learning process to make it more enjoyable and engaging, Game-based learning involves modifying a game to teach a specific skill or learning result, and Serious games, are ones created where education is the primary goal, rather than entertainment [11, 15, 33]. All of these three game-related mechanisms seek to make learning more interesting and inspiring [15]. Many studies have shown that utilizing gamified media in medical education may confer advantages, however most of these are virtual and computer-based [15]. A study conducted at the Glasgow Science Centre used a science communication app on 3D anatomy for public engagement activities involving skull anatomy and cycling helmet safety. They found that by integrating the fun and enjoyable elements of a game with educational objectives (gamification), players could learn complex scientific concepts through active exploration. They also proposed that the development and inclusion of serious games in public engagement activities would be a useful adjunct [36].

Mounting evidence suggests that educational games can provide an enjoyable way of learning, improve student engagement, stimulate the motivation of students to learn, and contribute to improvements in teaching outcomes [38]. Gamification can provide alternative approaches for educators and in most cases, these are well-received and can create an immersive experience, and are effective, engaging, easy to understand, interesting, and educational [38].

Educational games should never disrupt the learning process and when they are introduced into the classroom, it is important to incorporate them into the pedagogy [2]. We believe that students learning must align with learning objectives/outcomes and the 3D foot and ankle puzzle session had clear learning outcomes and was embedded in the curriculum. It has been proposed that educational games are able to create a social constructivist learning environment, where learners construct their knowledge by interacting with peers and instructors. This cooperative learning concept can support learners to achieve learning outcomes and promote social skills [3].

Dissection is often termed as destructive, rather than constructive process [22]. The disassembly process inherent in dissection does not allow for repeated explorations, particularly ones that are guided by different objectives. Similarly, it could be argued that a cadaveric foot skeleton (or skeleton model) could have been used in teaching, however these are usually wired or fixed. The advantage of using a 3D printed puzzle in teaching anatomy is that it allows the building up of the structures from its components which is constructive and creative and enhances the learning experience.

The effectiveness of combining 3D printed models and gamification in undergraduate medical education has not yet been investigated. This study aims to investigate if using a 3D printed foot and ankle puzzle, combing these two learning aids, can enhance the learning experience and be effective for teaching anatomy.

## Methodology

This study comprised two identical sessions one year apart with an entire cohort of 129 first-year medical students on a single MBChB programme at Aston University in the first session and the entire subsequent cohort of 138 first-year medical students. The entire cohort of first-year medical students on the MBChB programme were included and attendance was monitored. The first cohort consisted of 54 male and 75 female, and the ages ranged from 18 to 30 (average age 19.5 ± 1.6 standard deviation). 109 students were from the United Kingdom and 20 from overseas (18%). The second cohort consisted of 54 male and 84 female, and the ages ranged from 17 to 26 (average age 19 years ± 1 standard deviation). 110 students were from the United Kingdom and 28 from overseas (20%). Eligible participants were undergraduate medical students recruited from Year 1 studies. Inclusion criteria were age 18 or over, students enrolled in an undergraduate medical training program and were in their first year of study. Participation was voluntary, with written informed consent obtained, with 242 students participating in the study, 242 completing the pre-session questionnaire and 195 completing the post-session questionnaire.

### Design and printing of the 3D puzzle

Anonymised CT dataset for the foot was downloaded from Embodi3D^®^ (Available at: https://www.embodi3d.com/files/file/12930-foot-3d-scan/). Materialise Mimics 25.0 (Materialise, Leuven, Belgium) a clinically certified software was used to perform segmentation. To segment the foot an initial mask was created by setting the threshold value at 226–2145 Hounsfield Units to accurately delineate bone tissue. The axial, sagittal, and coronal planes were cropped to isolate the left foot only. The foot was divided using the split mask tool into the following anatomical components: Tibia, Fibula, Calcaneus, Talus, Navicular, Cuboid, Medial, Intermediate, and Lateral Cuneiform, Metatarsals, and Phalanges. Holes and cavities were filled (smart fill tool) to ensure a solid mask. The final segmented model was saved in STL file format and exported into Materialise 3-Matic 17.0 (Materialise, Leuven, Belgium) for post-processing and surface rendering. Spikes and roughness were removed (local smoothing tool) on the mesh surface and cavities for the magnets were created (pin holes connection function). Ultimaker Cura 5.2.2, an open-source software (Ultimaker, Utrecht, Netherlands) was used for slicing. The layer thickness was set to ‘fine’ with a resolution of 0.1 mm. Both the build and support material were printed using a 0.4 mm diameter print core (nozzle head). The default print speed was configured to 70 mm/s. The infill percentage was set at 20% to balance structural strength and material efficiency and the ironing function activated to achieve a smoother top layer. The parts were printed using an Ultimaker S5 printer (Ultimaker, Utrecht, Netherlands) through Fused deposition modelling (FDM), a printing technology in which thermoplastic filament is extruded layer by layer through a heated nozzle. The model material used was white Polylactic Acid (PLA) filament and water-soluble Polyvinyl Alcohol (PVA) filament for the support material. Following printing, parts were left in a centrifugal water bath to dissolve the PVA support material. To further smooth the surface and remove any visible layer lines, sanding was performed using a handheld rotating precision sander (Dremel, (FormLabs)) and manually using sandpaper of varying grit. Magnets were attached to the designed cavities of the foot model using standard epoxy resin super glue.

It is possible to scale up or down in size of the prints, for example, to show finer details but also if the original specimen is larger than the printer, to improve portability, or to reduce time to print and cost [17, 26]. One study recommended using full-scale models, as they found that having only access to “scaled” models, could lead to a lack of understanding of the real size and relations of the anatomical structure [26]. Our printed puzzle model had lifelike proportions in scale, making it easy to handle and realistic.

### Session format

The session was conducted in a familiar and neutral environment and the room was arranged to ensure there were no physical barriers or interruptions. At the start of the session, all participants were given 5 min to complete the questionnaire detailed in Table 1 (pre-session questionnaire). Participants were also given 5 min to complete the same questionnaire after the practical part of the session (post-session questionnaire). Responses were graded on a five-level Likert scale as follows:

- Not applicable.
- Strongly disagree.
- Disagree.
- Agree.
- Strongly agree.

**Table 1:** Questionnaire statements for student responses before and after the session

1	I feel confident in learning anatomy using models, compared to traditional methods such as diagrams.
2	I feel confident in applying my pre-existing knowledge of anatomy to identifying structures in the foot.
3	I feel confident in applying my pre-existing knowledge of the anatomy in identifying the calcaneus of the foot.
4	I feel confident in applying my pre-existing knowledge of the anatomy in identifying the talus of the foot.
5	I feel confident in applying my pre-existing knowledge of the anatomy in identifying the navicular of the foot.
6	I feel confident in applying my pre-existing knowledge of the anatomy in identifying the cuboid of the foot.
7	I feel confident in applying my pre-existing knowledge of the anatomy in identifying the cuneiforms of the foot.
8	I feel confident in applying my pre-existing knowledge of the anatomy in identifying the metatarsals of the foot.
9	I feel confident in applying my pre-existing knowledge of the anatomy in identifying the arches of the foot.
10	I feel confident in applying my pre-existing knowledge of the anatomy in identifying the joints of the foot and ankle.
11	I feel confident in applying my pre-existing knowledge of anatomy to enhance my ability to learn traditional physical examination skills of the foot.

**Fig. 1:**
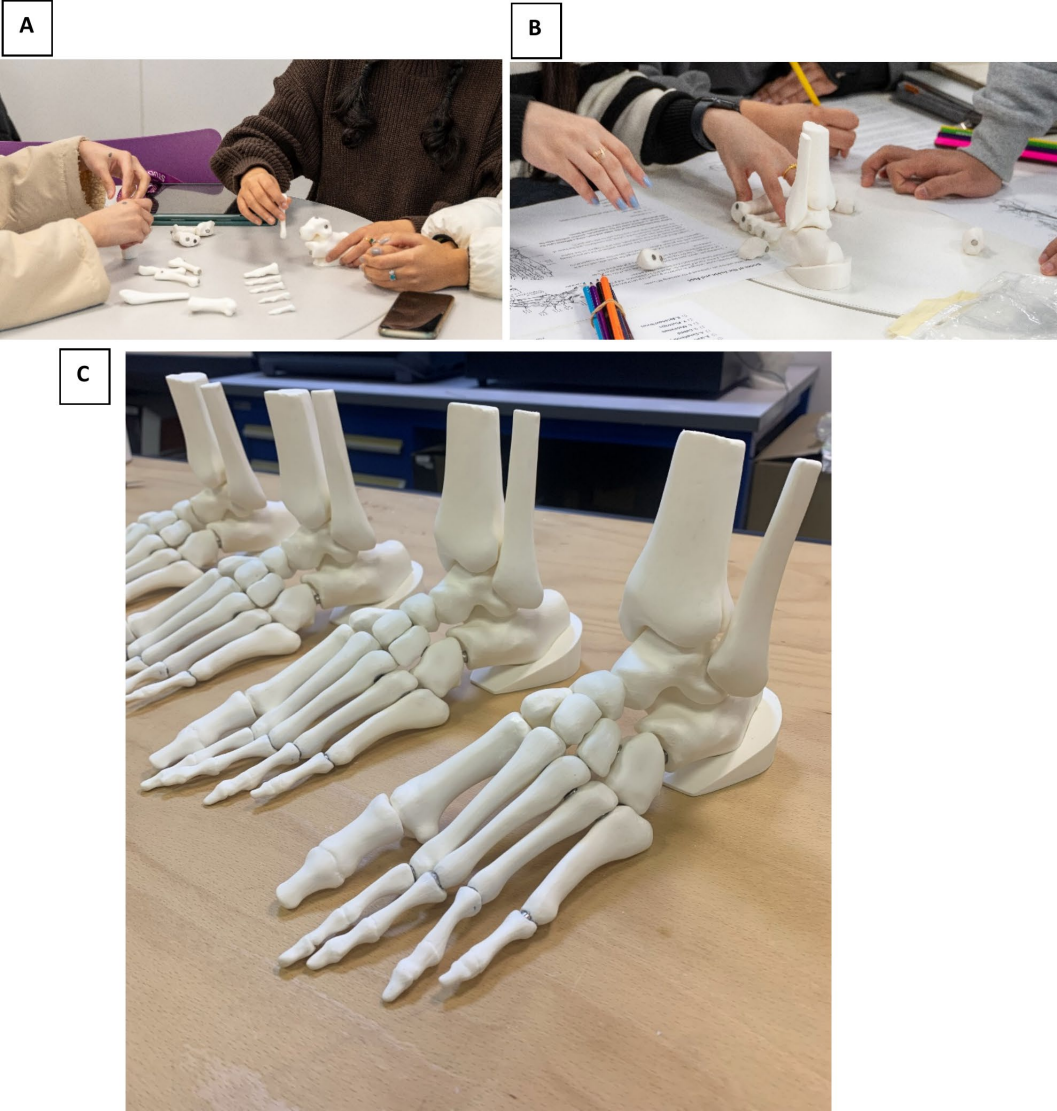
3D magnetic foot jigsaw being assembled (**A** and **B**) and completed (**C**)

Participants were allocated to small groups of up to 6 students per group and each group was given a 3D Printed Anatomy Puzzle to assemble. Figure 1 shows the model being assembled (Fig. 1A and B) and completed (Fig. 1C).

As a group, the aim was to put together the bones of the foot and ankle. Participants were given 5 min to familiarize themselves and attempt the puzzle without any aids. Participants were then provided with a colouring worksheet to use as an aid to complete the puzzle and were then given a further 15 min to complete the puzzle. Students knew there was a deadline to complete the puzzle before the end of the session and also that they were competing against other teams. To encourage friendly rivalry and competitiveness, a prize was promised to the fastest team to complete the puzzle correctly.

We believe gamification which includes competition among students could be beneficial and boost performance. Competition and collaboration in game-based learning can both motivate and engage learners. One study randomly assigned students into either a collaboration or competition group, in the latter students were required to solve the problems individually. They found a significant increase in the knowledge assessment scores in both groups, however the collaboration group showed significantly higher improvement. They argue that a social learning environment where learners can interact with their peers and instructors could support their learning, for example students who require assistance would benefit from constructivist collaboration [3]. In our study we combined both elements. Students worked together in groups, helping each other in a collaborative approach but there was also competition, but this was between the groups and not individuals.

Students were asked to provide comments on question 1 before and after the session.

### Quantitative data analysis

For both the pre-session and post-session questionnaires the number of responses for each of the questions on the Likert scale were tabulated in a contingency table. Pre-session and post-session contingency comparison of responses were compared statistically by Fisher’s exact test using GraphPad Prism 9.5.1.

### Qualitative data analyses

The participant comments to question 1 before and after the session was analysed by semantic deductive thematic analysis. The responses were coded by two clinical teaching fellows independently and then the codes were grouped into themes. The Kappa coefficient for the inter-rater reliability of the coding was 0.85. Data were anonymized by a senior faculty member. Statistical analyses were carried out using GraphPad Prism 9.5.1. The themes and codes analysed are detailed below:

- Theme 1: Engagement.
- The code of engagement was regarded as a separate theme.
- Theme 2: Models are a good aid to learning.
- Responses in this theme were coded as 1. good mental learning aids; 2. good physical learning aids.
- Theme 3: Students prefer 3D models to traditional methods of learning foot anatomy.

Responses in this theme were coded as follows: (1) preference for traditional 2D methods, such as labelling diagrams; (2) preference for models; (3) equal preference; (4) not enough experience to express a preference.

### Rigor

Quality of the written response data was ensured by the following methods: analyst triangulation was used to ensure data credibility with two analysts from different backgrounds (a senior lecturer and a junior doctor) independently coding the thematic analysis; a thick description of the study (see above); dependability and confirmability was ensured by an external audit by a researcher outside the Medical School. The wording of the questionnaires was selected to reduce the risk of bias into the study design. Specifically, the wording was based on a questionnaire in another peer-reviewed publication [19] that was modified according to the guidelines on reducing study bias [4]. Likert scales were chosen to study student self-assessment of their confidence in anatomy based on previous publications that show this is a good method for that specific exercise [5, 8]. A control group was omitted so that students should not be unfairly advantaged or disadvantaged in their learning due to their participation in research as recommended for explorative research [29]. Quantitative data was obtained in addition to qualitative data to minimise the effect of the lack of a control group [9].

## Results

### Quantitative analysis

Students had statistically similar opinions of 3D modelling before and after the session (Table 2, question 1). However, contingency analysis of the Likert scale questionnaires showed a statistically significant improvement in confidence in their knowledge of foot anatomy in all ten areas (Table 2, questions 2 to 11).

**Table 2:** Number of learner responses to each of the questions listed in the methods

Question	Pre- or post-test answer	Percent of total responses					Fisher’s exact test
		Not applicable	Strongly disagree	Disagree	Agree	Strongly agree	
1	Pre-test	0.00	1.65	11.16	52.89	34.30	*
	Post-test	0.51	1.54	3.59	43.08	51.28	
2	Pre-test	1.24	1.65	30.58	50.83	15.70	****
	Post-test	0.00	0.51	3.59	51.28	44.62	
3	Pre-test	1.24	3.31	22.73	50.41	22.31	****
	Post-test	0.51	0.00	2.56	48.72	48.21	
4	Pre-test	1.65	3.31	28.93	47.93	18.18	****
	Post-test	1.03	0.00	3.59	48.21	47.18	
5	Pre-test	0.83	4.58	33.75	46.25	14.58	****
	Post-test	1.03	0.00	8.21	44.10	46.67	
6	Pre-test	0.83	3.32	29.46	49.79	16.60	****
	Post-test	0.51	0.00	9.23	45.64	44.62	
7	Pre-test	0.41	3.73	32.37	45.23	18.26	****
	Post-test	1.04	0.52	6.77	48.44	43.23	
8	Pre-test	0.42	2.93	15.48	55.23	25.94	***
	Post-test	1.04	0.00	2.59	46.11	50.26	
9	Pre-test	0.83	5.00	22.92	53.75	17.50	****
	Post-test	0.52	0.52	5.67	53.09	40.21	
10	Pre-test	0.84	5.44	29.29	48.95	15.48	****
	Post-test	0.52	0.52	7.25	51.30	40.41	
11	Pre-test	1.24	7.05	28.22	49.79	13.69	****
	Post- test	0.52	0.52	6.70	50.52	41.75	

Contingency comparisons of the responses before and after the session were made by Chi-square test for trend. * *P* < 0.05, *** *P* < 0.001, **** *P* < 0.0001

### Qualitative analysis

#### Theme 1: Engagement

Following the session there were 14 responses that expressed how “fun and interesting and engaging” the 3D model session was (15% of all post-session responses). The words “stimulating” and “engaging” appeared 4 times each in these 14 responses (the word “fun” appeared the most times at 5). See Supplementary Table 1 for full responses in Theme 1. There were no pre-session responses that stated enjoyment of using models, indicating that the students did not expect to be engaged by the foot anatomy session, even though a majority expressed a pre-session preference for 3D models.

#### Theme 2: Models are a good aid for learning

##### Code 1: The 3D foot models are a good aid to remember the foot anatomy

7 post-session responses stated that the 3D models helped the students visualize foot anatomy (7% of total post-session questionnaires). The post-session responses also added to this by stating that the 3D models might help them remember the foot anatomy, as well as visualize it. See Supplementary Table 2 for full responses in Theme 2 Code 1. 11 pre-session responses stated that the 3D models helped the students visualize foot anatomy (9% of total completed questionnaires). The students expected the session to help them visualize the anatomy of the foot, and this was evidenced by the session. However, the students had not expected the 3D models to help them remember the anatomy.

##### Code 2: The tactile nature of the 3D models helped learn the physical structure of the foot

12 post-session responses stated that the physical handling of 3D models helped the students appreciate the physical anatomy of the foot (11% of the total completed questionnaires). This is like the pre-session responses, when 10 responses stated that the physical handling3D models helped the students appreciate the physical anatomy of the foot (10% of the total completed pre-session questionnaires). The students expected that the “hands-on” experience would help and the post-session responses supported this expectation, giving additional reasons as to why this would be so: e.g., “physically holding the part of the body and manipulating it in the hand makes it easier to understand the parts of the structures”; “actually holding and looking at models of the bones really reinforced my knowledge of the bones in the foot and ankle joint”. See Supplementary Table 3 for full responses in Theme 2 Code 2.

#### Theme 3: Students prefer 3D models to traditional methods of learning foot anatomy

Of the post-session responses that expressed a preference, 100% preferred 3D models to more traditional learning aids, although only 4 post-session responses were recorded. This confirmed the students’ expectation that the majority would prefer the 3D models, when 23 (54%) pre-session responses indicated a preference for more 3D models for learning anatomy over more traditional methods. Only 7 (16%) pre-session responses indicated a preference for more traditional methods of learning anatomy over 3D models, and 7 (16%) also responded with no preference. 6 (14%) pre-session responses indicated not enough experience with 3D models to compare their effectiveness in learning anatomy to more traditional methods. No post-session comments reflected on a lack of experience with models.

The predominant reason for preferring traditional methods of teaching anatomy, was that the students felt that diagrams are “more simplistic” and “clearer to understand”. However, the predominant reason for preferring 3D models was not the direct opposite of the reason for preferring diagrams, i.e. less “simplistic” or more detailed. Rather, the students related to 3D models as they felt they were more “realistic” than diagrams. See Supplementary Table 4 for full responses in Theme 3 Code 1: Prefer traditional methods, Supplementary Table 5 for full responses in Theme 3 Code 2: Prefer models. The predominant reason for expressing an equal preference for 3D models and traditional methods of teaching foot anatomy was a positive reflection that they are both useful aids and that they complement each other. For example, “mix of both is preferred” and “traditional methods are more useful with models”. See Supplementary Table 6 for full responses in Theme 3 Code 3: equal or no preference, and Supplementary Table 7 for full responses in Theme 3 Code 4: Not enough experience to express a preference.

## Discussion

This study has confirmed that 3D-printed models can be produced appropriately and if combined with gamification, can enhance the learning experience and be effective for teaching anatomy. 3D printing is generally accepted to be low cost [16, 17, 23, 41], and combined with the open access digital databases, more 3D printing will be brought to the forefront of classrooms [23]. 3D printed reproductions are highly accurate when compared to the original specimens, durable, suitable for rapid reproduction, reproducible, and offer the advantage that they can be handled in a classroom environment [1, 7, 17, 41]. Cost-effective in-house printing could be of particular benefit to locations where the “real thing” is unavailable and there are financial restraints [1, 7]. As they are not actual human tissue, they avoid any cultural, ethical and health and safety issues associated with viewing cadaver specimens [1, 16, 17, 41]. In-house printing also means that anatomical models can be customized to fit pre-existing lesson plans [23]. A key component of students learning is that activities, even games, align with learning objectives [15] and this is why our 3D printed foot and ankle model, and its gamification, was embedded in the curriculum, so that the activity was meaningful and worthwhile for the students. The session followed learning outcomes which aligned those of the previous week’s lectures. The pre-test and post-test questionnaire responses demonstrated that 3D-printed foot and ankle puzzle can be used as an effective teaching aid, that brings about an increase in student confidence in foot and ankle skeletal anatomy knowledge. This finding supports previous reports that found enhanced anatomy learning in a randomized control trial in the group that only used 3D-printed material, compared to those that used only cadaveric or combined materials [16]. The authors explain this beneficial effect of 3D prints over cadaveric materials by stating that there is evidence that novice students exhibit significant apprehension, stress, and anxiety with cadavers, that there is a tendency for students to avoid contact with cadavers, and that students are more willing to handle 3D prints and plastic models when compared with real cadaveric material [16].

The present 3D printed puzzle model study demonstrated a strong positive tactile element that the students found beneficial to the learning experience. This agrees with previous studies that found printed bony models are a reasonable alternative to cadaveric specimens [41], unlike printed models of soft tissues which do not have adequate haptic and colorific quality [17]. 3D printed models have been proposed as an effective method for manufacturing human anatomical specimens in response to the shortage of cadaver specimens and the poor simulation results of anatomical specimen substitutes [10, 28, 41].

Despite the advances in 3D modelling in teaching anatomy, the question whether 3D printed models truly improve learning remains unanswered [10]. The themes in the pre- and post- session questionnaires are similar. Before the session, the majority opinion was that 3D models would be beneficial both as a visual and as a tactile aid for effective learning. This opinion was consolidated by the post-session comments. After the session, there were many comments saying how enjoyable and engaging the 3D models were. Games are well known to help learning by creating memories and experiences, and for their potential to improve student interaction and engagement [3, 20]. Educational game benefits include providing opportunities for active learning, combining other learning activities (such as feedback, testing, and spaced repetition), autonomy, positive experiences for students, immersive experiences and improving study outcomes [38]. It may allow students to gain knowledge and skills from their mistakes. Failure within the game activity will mean learners, through a form of feedback, adapt their strategy so that they can complete the task [3].

Gamification is the application of game concepts (e.g. points, leader boards, prizes) to the learning process to make it more enjoyable and engaging [15]. The evidence for gamification in the educational setting is increasing and the idea is gaining momentum in the medical field [15, 18, 20, 33]. Reported benefits of gamification include making learning fun, improving motivation, being able to use incentives and rewards, improving understanding and memory, sustainable use of resources, using technology, and allowing experiential learning, feedback, repetition, competition, collaboration, and diversity [15]. In our study, the gamification of the 3D model provided a puzzle element to the session, where the students were challenged mentally on their anatomical knowledge of the individual parts and their ability to put it all together to replicate the real anatomy of the ankle and foot. The combined elements of the puzzle and the features of the 3D model assembly made the activity fun and conducive to active learning. The possibility of having fun during the anatomy session was not something our students had considered before the session. Gamification also appears to promote increased engagement [15]. In our study, even though the number of completed questionnaires dropped from 242 before the session to 195 after the session, the level of engagement was still very high. Moreover, while the specifics may differ, the principles of gamification remain relevant for complex anatomy and that educators can adapt gamification strategies to address more advanced anatomical concepts [25].

Engagement may have been promoted by the groups competing against each other, with the fastest team being rewarded with a prize. This would agree with a study which found that these competitions based on gamification were favourably received, fostered enthusiasm and enhanced motivation in trainees and teachers [18]. The idea of gamification is generally embraced by participants and satisfaction rates are high [2, 33], which is similar to our findings. Gamification has also been found to help mitigate the stresses associated with anatomy education [2, 20]. By lightening the mood and environment, active user participation and effective learning and consolidation can take place [2, 20].

### Limitations

One limitation of the study was that students were not divided into a testing and control group, for example, a group could have used a conventional wired foot and ankle skeleton (“no puzzle / game” group) and/or a group could have had no 3D model at all (“no 3D model”). However, we did not do this, as we wanted the entire cohort to benefit from the resources and novel teaching method.

To enhance the rigour of the study design the responses were anonymous [12]. This meant that, although we had a subjective student score based on their confidence in identifying the anatomy, it could not be compared with their actual academic assessment performance, for example evaluated by pre-and post- session knowledge assessments. Several studies have shown that gamifying anatomy education, succeeded in producing better academic grades, probably by increased engagement, collaboration, and swift feedback [2, 3].

## Conclusion

This is the first study to test the effectiveness of combining 3D printed anatomy models and gamification in undergraduate medical education. We found that combing these two learning aids can enhance the learning experience. We would therefore recommend using 3D-printed puzzles as an effective and engaging way to teach and learn anatomy.

### Supplementary Information

Below is the link to the electronic supplementary material.

Supplementary file1 (DOCX 3603 KB)

## Data Availability

No datasets were generated or analysed during the current study.
