# Supplementary material for: Use of 3D foot and ankle puzzle enhances student understanding of the skeletal anatomy in the early years of medical school
Source: Surg Radiol Anat. 2024 Jul 26;46(9):1429–38. doi: 10.1007/s00276-024-03439-1 (PMC11322274; doi:10.1007/s00276-024-03439-1)
Supplement: Supplementary file 1 — Supplementary file1 (DOCX 3603 KB) [file 276_2024_3439_MOESM1_ESM.docx]

**Appendices**

**Supplementary table 1:** Theme 1 full responses

*Post-session responses:*

Very brain stimulating.

I am a visual learner, and this was a great interactive way of learning anatomy. I really liked this.

Was fun and interesting and engaging

Was fun

Very engaging and stimulating way of learning

It’s a lot more engaging than using diagrams

It was interesting and fun

Fun

It was good

Very fun and stimulating

Absolutely amazing please do more

An amazing activity

Stimulating and fun

Engaging

**Supplementary table 2:** Theme 2 Code 1 full responses

*Pre-session responses:*

Helps me realise what it looks like

Just help visualising when it’s 3d

Models allow you to visualise

3D models allow better visualisation

i find it really useful to learn in 3d to understand the actual position of things in the body

it’s easier to visualise when using real life 3d models

Models provide a visualisation of where structures are

Models are important to visualize in 3D

I feel it helps more to visualise anatomy

Helps to visualise anatomy more easily

Models help more as a visual aid.

*Post-session responses:*

It’s easier to visualise

Easier to understand structures in 3D format

Helpful to visualise

It helped me to visualise the foot well

Easier to visualize

Helped 3D understand

Helps with retention and better aids understanding of how things fit together

**Supplementary table 3:** Theme 2 Code 2 full responses

*Pre-session responses:*

I like seeing the anatomy models so I can see where muscles, bones and ligaments are and relate it to an actual patient

Helps with real-life practice, as we’re not going to be operating on diagrams

I believe it will help when it comes to clinical skills

Models being 3D help understanding of location

Visualising the anatomy in 3D and actually being able to touch everything is helpful

Models are much more beneficial to help me learn because they are in 3D and allow much better differentiation between structures (diagrams are limited in this)

I like using the models as i am able to physically see the areas if the body, this then helps me to remember and learn the inform

Benefit from being able to see and touch

Models put everything into a 3d perspective

I prefer hands on learning experience

I prefer hands on experience.

Good practical way of learning to visualize better.

*Post-session responses:*

Very useful

Was good having 3D and being able to take apart

Feeling the pieces on 3D allows it to truly be learnt

It was good and tangible.

Physically holding the part of the body and manipulating it in the hand makes it easier to understand the parts of the structures.

Helped me understand the relative positions of the bones

Visual representation and seeing how things fit together

More practical and realistic, understood the 3d aspect

It was very helpful in understanding the shapes and names of the bones and how they fit together

Actually holding and looking at models of the bones really reinforced my knowledge of the bones in the foot and ankle joint.

**Supplementary table 4:** Theme 3 Code 1 full responses

Code 1: Prefer traditional methods.

Pre-session responses:

Prefer traditional methods

I prefer diagrams simply because they're all very similar.

Easier to visualise in 2d than 3d

I find models hard

Diagrams help to place features better in context to other landmarks, more simplistic

I feel that diagrams are clearer and easier to understand

It’s very hard to find motivation to actually start with models

Post-session responses:

There were no post-session responses that stated a preference for traditional methods.

**Supplementary table 5:** Theme 3 Code 2 full responses

Code 2: Prefer models

*Pre-session responses:*

I feel confident in learning anatomy models compared to traditional methods, e.g. diagrams.

Easier when it’s 3D

Models are good

Visualising the position of muscles and tendons are challenging with traditional methods.

I feel confident in learning anatomy models compared to traditional methods, e.g. diagrams.

It’s helpful to see anatomy via a 3D representation.

Visual form of learning is more helpful *(N.B. assuming the visual form referred to is the model)*

I enjoy the 3d nature

Visual learning with anatomy helps to put things into perspective

3D therefore allows for better understanding

Models help

I find the 3D models in tutorials useful so far.

Using models makes it much easier to see the placement

I think it’s better as the human body is viewed from different perspectives

A lot easier to learn anatomy in real life rather than from a book

I like using models as the learning experience seems more realistic

Can visualise easier with models

Models can be easier to use than diagrams as you get a better idea of the 3D space

Models are more realistic/accurate visual representation

Understanding is alot better when using 3D models than 2D models

Images don’t always help with appreciating the 3D anatomy

I find using anatomy modes helps me in comparison to looking at 2D structures

‘3d’ diagrams are still 2d so the 3d models are much more realistic and interesting to use

*Post-session responses:*

4 (100 %) post-session responses indicated a preference for more 3D models for learning anatomy over more traditional methods.

Much easier to learn this way

Yes, I feel confident in learning anatomy models compared to traditional methods

It allows for a new type of learning beyond memorisation

Found it very useful more to learn anatomy rather than doing worksheets.

**Supplementary table 6:** Theme 3 Code 3 full responses

Code 3: equal or no preference

*Pre-session responses:*

Mix of both is preferred

Both are fine

I sort of agree I still need to review the lectures in order to comment on the other questions

Traditional methods are more useful with models

I’m ok so far but I need to do an exam to see how my studies have been working

i think u need a combo

Consolidates learning

*Post-session responses:*

There were no post-session responses that stated an equal preference.

**Supplementary table 7:** Theme 3 Code 4 full responses

Code 4: not enough experience to express a preference

*Pre-session responses:*

Not sure I’ve had enough exposure to models yet to be able to compare

I do not know how to learn anatomy at all.

I’m not sure, have not really used models before

We have had little experience with models

I have not used models much, so do not feel confident using them to identify smaller features.

Need more experience using models but would prefer it.

*Post-session responses:*

There were no post-session responses that stated not enough experience of methods.
